# Functional recovery differences after stroke rehabilitation in patients with uni- or bilateral hemiparesis

**DOI:** 10.17712/nsj.2017.3.20170010

**Published:** 2017-07

**Authors:** Saad M. Bindawas, Hussam M. Mawajdeh, Vishal S. Vennu, Hisham M. Alhaidary

**Affiliations:** *From the Department of Rehabilitation Sciences (Bindawas, Vennu), King Saud University, and Rehabilitation Hospital (Mawajdeh, Alhaidary), King Fahad Medical City, Riyadh, Kingdom of Saudi Arabia*

## Abstract

**Objective:**

To examine the functional recovery differences after stroke rehabilitation in patients with uni- or bilateral hemiparesis.

**Methods:**

In this retrospective study, we included data from the medical record of all 383 patients with uni- or bilateral hemiparesis after stroke who were admitted to King Fahad Medical City-Rehabilitation Hospital between 2008 and 2014 in Riyadh, Kingdom of Saudi Arabia. According to the site of hemiparesis, we classified patients into 3 groups: right hemiparesis (n=208), left hemiparesis (n=157), and bilateral hemipareses (n=18). The patients (n=49) who did not have either site of hemiparesis were excluded. The Functional Independence Measures (FIM) instrument was used to assess the score at admission and discharge. A post hoc test was conducted to examine the functional recovery differences between groups. Multiple regression analyses were used to confirm the findings.

**Results:**

Amongst the three groups, there were significant (p<0.05) differences in the total-FIM score as well as motor- and cognitive-FIM sub-scores between admission and discharge of stroke rehabilitation. The differences were significantly greater in the bilateral hemipareses group than in either unilateral hemiparesis group. Multiple regression analyses also confirmed that the site of hemiparesis significantly (p<0.05) differs in the total-FIM score as well as motor-FIM and cognitive-FIM sub-scores.

**Conclusion:**

Our results demonstrate that differences in functional recovery after stroke rehabilitation may be influenced by the site of hemiparesis after stroke.

Hemiparesis described as complete or incomplete muscular weakness or paralysis affecting either side of the body after a stroke.^1^ This condition stands out as the second most frequent and widely recognized impairment that had been reported in approximately 65% of patients.^2^ When this condition happens, the patients may show associated dysfunctions with either or both sides of the body that can make difficult performing essential activities of daily living (ADLs).^3^ These associated dysfunctions may occur because of cutting off blood supply (ischemic stroke) or bleeds (hemorrhagic stroke), which damage that area of brain tissue.^4^ To improve functions, blood supply, or stop bleeds, the patients need rehabilitation after stabilization, usually 24-48 hours of being hospitalized.^5^

The brain is a highly complex organ that influences different body functions.^6^ If stroke happens at the left side of the brain, the right side of the body has hemiparesis. These patients may have difficulty in talking and understanding (Aphasia),^7^ and also difficulty in determining left from right. While if stroke happens at the right side of the brain, the left side of the body has hemiparesis. These patients may have trouble in coordinating movement (ataxia),^8^ which leads to loss of balance and difficulty in walking.^9^ Although, if a stroke happens in the brain stream, it can influence both sides of the body and may leave somebody in a ‘secured’ state. At the point when acquired state happens, the patient is generally unable to talk or accomplish any development beneath the neck.^4^ Just as strokes have variable effects based on an interruption of blood flow or bleeds in the brain sites, the course of functional recovery (FR) from stroke in the inpatient rehabilitation may also vary.^10^ Findings from the published study have reported different outcomes of FR after stroke rehabilitation among patients with right or left hemiparesis.^8^,^11^ This has prompted deep confusion and instability about the clinical significance and the importance of differences in FR after stroke rehabilitation among patients with uni- or bilateral hemiparesis. Therefore, there may remain a need for data on differences in FR among patients with uni- or bilateral hemiparesis in Saudi Arabia.

Despite the presence of intensive stroke rehabilitation programs in Saudi Arabia, there remain limited data discussing the functional outcomes in patients with uni- or bilateral hemiparesis after stroke.^12^,^13^ This lack of knowledge regarding Saudi Arabian healthcare resources highlights the need to examine the differences in FR after stroke rehabilitation among patients with uni- or bilateral hemiparesis due to stroke.^14^ This serves to address country-specific cultural and social factors that may influence FR in this group of patients. Therefore, this study aims to examine the FR differences after stroke rehabilitation in patients with uni- or bilateral hemiparesis.

## Methods

This retrospective study received ethical approval by the Committee on Human Research, the Institutional Review Board at King Fahd Medical City-Rehabilitation Hospital (KFMC-RH), Riyadh, kingdom of Saudi Arabia (approval numbers: 14-273).

### Design and Setting

The electronic medical record database review was conducted by 2 of the present investigators at the KFMC-RH. The time frame between 2008-2014 was determined based on the availability of medical records by stroke diagnosed according to the International Classification of Diseases, Ninth Revision (ICD-9) codes 348-438 and 799.3. Differences of opinion regarding medical records were resolved by discussion between the investigators until consensus was reached. The KFMC-RH is the largest tertiary inpatient rehabilitation hospital in Riyadh, Kingdom of Saudi Arabia. The hospital follows the Uniform Data System for Medical Rehabilitation (UDSMR) protocol when administering the FIM scale.

### Participants

We included data of 383 patients with uni- or bilateral hemiparesis after stroke who were treated at the KFMC-RH stroke rehabilitation program between 2008-2014. Based on the site of hemiparesis, we classified patients into three groups: right hemiparesis (n=208), left hemiparesis (n=157), and bilateral hemipareses (n=18). The patients (n=49) who did not have either site of hemiparesis were excluded.

### Outcome measures

The FIM was used to score the level of assistance required for a patient to accomplish ADL.^15^ The FIM contains 18 items, including 13 motor tasks and 5 cognitive tasks. Each work is given an ordinal score between 1-7, with the minimum score of 1 representing complete dependence on others for the task; and the maximum score of 7 representing full independence. Accordingly, the total FIM score ranges from 18 to 126, with higher scores reflecting greater independence. Functional Independence Measures scores were measured sequentially at admission and discharge to determine the effects of therapy. The reliability and validity of the FIM instrument for patients with stroke are well-established.^15^

### Statistical analysis

Frequencies were presented as percentages (%) for categorical measures, and continuous measures were presented as means (standard deviations). For group comparisons, a chi-square test was conducted for frequencies, a post hoc analysis (ANOVA) was performed for continuous variables and for analyzing the differences in FR.^16^ Multiple regression analyses were used to magnitiude the differences of FR between the the site of hemiparesis.^17^ Statistical analysis was performed with SAS software, version 9.2 (SAS Institute, Inc, Cary, NC).^18^

## Results

**Figure 1:**
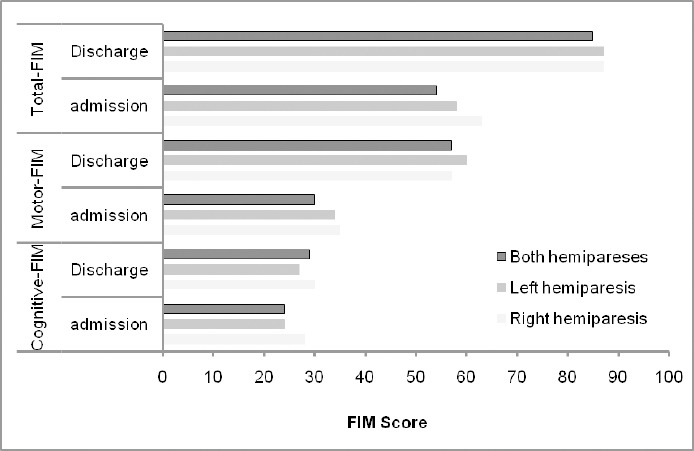
Deference in functional improvement scores after stroke rehabilitation in patients with uni- or bilateral hemiparesis.

**Table 1** shows descriptive characteristics of all patients in the 3 groups. The mean patient age was 54.9±15.3 years, and just over half of the patients were male (56%). Patients with bilateral hemipareses were 11 years younger (age 48 years) than patients with right hemiparesis (age 59 years) and were 10 years younger than patients with left hemiparesis (age 58 years). The usual length of hospital stay was 47.5 days. The majority of patients with uni- or bilateral hemiparesis were discharged to home (98%). Among the 3 groups, functional score improved on total-FIM as well as motor-FIM and cognitive-FIM subscales after discharge from inpatient rehabilitation facility (**Figure 1**).

**Table 1:** Demographic characteristics of all patients, stratified according to the site of hemiparesis.

Variables	ALL N=383	Right hemiparesis 208 (54%)	Left hemiparesis 157 (41%)	Bilateral hemipareses 18 (5%)	*P*-Value
	mean±SD, n (%)		
**Age in years**	54.9±15.3	58.8±16.4	57.9±14.8	47.9±14.7	0.019
***Gender***
Male	213(56)	124(60)	82(52)	7(39)	0.13
Female	170(44)	84(40)	75(48)	11(61)
***Discharge disposition***
Home	374(98)	204(98)	153(98)	17(94)	0.61
Not to home	9(2)	4(2)	4(2)	1(6)
LOS in days	47.5±28.7	47.5±32.1	48.9±28.4	59.6±37.5	0.28

FIM - functional independence measure, LOS - length of stay

**Table 2** shows outcome measures for the 3 groups after stroke rehabilitation in terms of total-FIM as well as motor-FIM and cognitive-FIM sub-scores. Among the 3 groups after stroke rehabilitation, 3 were significant differences between admission and discharge (*p*<0.05) in total-FIM improvement (right hemiparesis: 23.9±15.7; left hemiparesis: 29.4±16.3; bilateral hemipareses: 31.5±21.9; *p*=0.003), motor-FIM sub-scores (right hemiparesis: 21.9±15.3; left hemiparesis: 26.2±15.1; bilateral hemipareses: 26.7±18.1; *p*=0.023), and cognitive-FIM sub-scores (right hemiparesis: 2.0±3.3; left hemiparesis: 3.2±3.9; bilateral hemipareses: 4.8±6.1; *p*=0.005).

**Table 2:** Outcome measures, stratified according to the site of hemiparesis.

Variables	Right hemiparesis N=208 (54%)	Left hemiparesis 157 (41)	Bilateral hemipareses 18 (5)	*P*-Value
	n (%), mean±SD		
Change in motor FIM	21.9±15.3	26.2±15.1	26.7±18.1	0.023
Change in cognitive FIM	2.0±3.3	3.2±3.9	4.8±6.1	0.0005
Change in total FIM	23.9±15.7	29.4±16.3	31.5±21.9	0.003

FIM - functional independence measure

**Table 3** shows multiple regression analyses between site of hemiparesis and FR. The table confirms that site of hemiparesis signficantly differs in total-FIM score (right hemiparesis: β=5.32, SE=1.71, *p*=0.002; left hemiparesis: β=5.65, SE=1.70, *p*=0.001; bilateral hemipareses: β=9.15, SE=3.63, *p*=0.012), motor-FIM sub-scores (right hemiparesis: β=4.22, SE=1.61, *p*=0.009; left hemiparesis: β=4.40, SE=1.60, *p*=0.006; bilateral hemipareses: β=6.59, SE=3.42, *p*=0.05), and cognive-FIM sub-scores (right hemiparesis: β=1.10, SE=0.39, *p*=0.006; left hemiparesis: β=1.25, SE=0.39, *p*=0.001; bilateral hemipareses: β=2.56, SE=0.83, *p*=0.002) after adjustment for age, gender, and LOS. The full adjusted model shows an explained variance (R^2^) of 0.029 for total-FIM, 0.015 for motor-FIM, and 0.034 for cognitive-FIM.

**Table 3:** Multiple regression analyses between site of hemiparesis and functional recovery.

Site of hemiparesis	Motor FIM			Cognitive FIM			Total FIM		
	β	SE	*p*-value	β	SE	*p*-value	β	SE	*p*-value
Constant	25.94	3.41	<.0001	2.88	0.83	0.0006	28.82	3.62	<.0001
Right	4.22	1.61	0.009	1.10	0.39	0.006	5.32	1.71	0.002
Left	4.40	1.60	0.006	1.25	0.39	0.001	5.65	1.70	0.001
Bilateral	6.59	3.42	0.05	2.56	0.83	0.002	9.15	3.63	0.012
Adj R^2^		0.015			0.034			0.029	

FIM - functional independence measure, adjusted for age, gender, and LOS

## Discussion

The objective of this study aims to examine the FR differences after stroke rehabilitation in patients with uni- or bilateral hemiparesis. Our results reveal that there is a significant difference in FR by the site of hemiparesis after stroke rehabilitation. Patients with bilateral hemipareses had significantly greater FR after stroke than patients with unilateral hemiparesis. Additionally, patients with left hemiparesis exhibited significantly higher FR than patients with right hemiparesis. In summary, overall outcomes indicated significantly different FR after stroke rehabilitation in patients with uni- or bilateral hemiparesis due to stroke. The magnitude of change was significantly higher for patients with bilateral hemipareses.

Our findings also showed significant differences between admission and discharge in total-FIM as well as motor-FIM and cognitive-FIM sub-scores in patients with uni- or bilateral hemiparesis. Compared to patients with bilateral or left hemiparesis, patients with right hemiparesis had the lowest magnitude of FIM score change. This finding was constant with the results of the similar study, which described that patients with left hemiparesis had significantly greater FIM score improvements than patients with right hemiparesis.^19^ These results from our study further corroborate the results of other studies discussing FR in patients with bilateral hemipareses after stroke.

In contrast, another study reported that patients with left hemiparesis had more severe FR than patients with right hemiparesis after stroke rehabilitation.^8^ A recent observational study was conducted in Italy to investigate post-stroke motor FR difference in the inpatient rehabilitation using the 13 motor items of the FIM instrument among patients with right or left hemiparesis.^8^ The outcomes of this study showed that the patients with right hemiparesis had more FR than patients with left hemiparesis. Thus, the site of hemiparesis effects the degree of recovery after stroke. Differences in research methodologies, such as type of patient, the measures used, the outcome criteria, cultural differences or age differences, may explain some of the variations in our study.^12^

In the present study, patients with bilateral hemipareses were younger (by 10 years or more) than patients with unilateral hemiparesis. Additionally, patients with bilateral hemipareses had greater FIM scores. Furthermore, patients with left hemiparesis had higher FIM score improvements than patients with right hemiparesis after stroke rehabilitation. This finding was related to that of the previous study conducted in Austria^20^ that revealed the effects of age and laterality of stroke as predictors of FR in patients with hemiparesis after stroke.

Also, our findings indicate that differences in the total-FIM score, motor-FIM, and cognitive-FIM sub-scores all had thresholds above which they could be considered clinically significant. This limit is called the minimum clinically significant difference (MCID). A recent study in Saudi Arabia reported that the change in total-FIM was not clinically significant (the MCID was 20).^21^ Our work differs from the finding of the latter study in that the total-FIM as well as motor-FIM, and cognitive-FIM sub-scores were all clinically significant. These results, therefore, have the potential to help physicians in Saudi Arabia interpret changes in FIM scores regarding their clinical significance.

In acute care, the physician has the established goal of significantly improving the blood flow or break up blood clots in the area of damaged brain tissue through medications (antithrombotics and thrombolytics) to stabilize the patient’s health status.^22^ After the patient is stabilized, usually 24-48 hours of being hospitalized, according to clinician decision, the patient will transfer into an in-patient rehabilitation facility to regain as much function as possible.^23^ In the in-patient rehabilitation, physical or occupational therapists aim to improve functions through a specialized rehabilitation program.^24^ Literature from published studies also demonstrate that a patient with a stroke who received treatment in in-patient rehabilitation is more likely to be functionally recover.^25^-^28^ Therefore, the findings of this study have important implications for the development of rehabilitation services with a professional team in Saudi Arabia for achievement of better functional outcomes, particularly in patients with bilateral hemipareses after stroke.^27^

The strengths of our work were as follows: first, KFMC-RH is a large tertiary in-patient rehabilitation hospital in Riyadh, Saudi Arabia, and is certified to use the FIM instrument. Second, this is the first study in Saudi Arabia that examined the differences in FR after stroke rehabilitation in patients with uni- or bilateral hemiparesis. The limitations of this study include its retrospective design and small sample size; larger studies may produce more accurate results. Additionally, the results are not generalizable as the study did not include all tertiary rehabilitation hospitals in Riyadh. Additionally, we have not addressed the manner of stroke onset and stroke severity that influences the FR of stroke rehabilitation. Another limitation is the deficiency of data regarding health covariables (for example, comorbidities, depressive symptoms, and BMI), as well as some sociodemographic information (for example, marital condition, education, living status, and race) that may affect FR.

In conclusion, this study demonstrates that differences in FR after stroke rehabilitation may be influenced by the site of hemiparesis after stroke. Patients with bilateral hemipareses may need a special intensive rehabilitation compared with patients having a right or left hemiparesis. Future large-scale studies at national level tertiary rehabilitation hospitals and studies exploring sociodemographic and health variable effects may provide better insights into FR among patients with hemiparesis. It may, in turn, help justify the establishment of more rehabilitation centers in Saudi Arabia.
